# Trigger Site Inactivation for the Surgical Therapy of Occipital Migraine and Tension-type Headache: Our Experience and Review of the Literature

**DOI:** 10.1097/GOX.0000000000002507

**Published:** 2019-11-12

**Authors:** Edoardo Raposio, Nicolò Bertozzi

**Affiliations:** From the Plastic Surgery Unit, Department of Medicine and Surgery, University of Parma, Italy.

## Abstract

Literature from the last decade has shown a correlation between resection of the occipital muscles and vessels and relief from migraine and tension-type headaches.

**Methods:**

The aim of this article was to describe the authors' technique to treat occipital migraine, while comparing our approach with the other currently available surgical options. Relevant anatomical issues and their implications in the surgical treatment of occipital migraine have been reviewed. We undertook a modified version of the currently used method of occipital migraine surgery. Patients completed questionnaires before and after surgery, and results were compared.

**Results:**

To identify all trigger points, we used a constellation of symptoms referred to by the patient rather than injection of botulinum toxin type A. The entire procedure was carried out under local anesthesia. In most of the patients (56) in whom a dilated/aneurysmal occipital artery was found, the procedure was limited to ligation of the occipital artery, with no further undermining of muscles or neurolysis, which reduced the invasiveness of the procedure.

**Conclusions:**

The main differences between our procedure and the currently used method were that (1) extensive undermining and muscular or nerve resection were not necessary and (2) no flap was transposed with the purpose of covering isolated nerves. Hence, our method could improve the currently used method, while minimizing its invasiveness.

## INTRODUCTION

Migraine headache (MH) is a common disorder affecting over 324.1 million people worldwide that can seriously decrease life quality and work productivity of the patients.^1^ It is estimated that in the United States 37 million people suffer from MH that is equal to 12% of the total population.^2^ The situation is similar in Europe, where more than 50% of adults reported at least 1 episode of headache during the last year and about 4% of the total population suffers from chronic headache.^3^ Furthermore, some studies indicate that the headache prevalence is increasing during the last decades in Europe.^3^

MH represents also a social and economic burden. In the United States, the total cost for MH treatment accounts for $13 to $17 billion each year and 112 million of missed workdays with an indirect loss of $13 billion.^4^

Despite its prevalence and debilitating nature, MH is still widely undiagnosed and undertreated.^4^ The first approach is usually a combination of pharmacologic treatments (both abortive and preventive drugs) and nonpharmacologic interventions such as behavioral and lifestyle changes.^4^ Despite all the available conservative options, a quite relevant group of MH patients remains refractory and does not achieve a satisfactory relief.

The idea to treat MH refractory patients with a surgical procedure is relatively recent. In 1999, Guyuron first described the elimination or improvement of MH in a group of cosmetic patients who underwent corrugator supercilii muscle resection for forehead rejuvenation surgery. One year later, Guyuron confirmed his hypothesis with a retrospective study showing the association between corrugator supercilii muscle resection and disappearance or reduction in MH symptoms.^5^

In recent years, further evidences and anatomical studies were determinant to validate the surgical approach. Although the pathophysiology of MH remains a matter of discussion, it seems evident that nerve compression in some specific areas plays a pivotal role in triggering MH episodes. More specifically, 4 main trigger zones have been described to be addressed by surgical procedures: frontal (site I: supraorbital and supratrochlear nerves), temporal (site II: zygomatic–temporal branch of the trigeminal nerve), endonasal (site III: trigeminal end branches), and occipital (site IV: great occipital nerve).^6^ The auriculotemporal nerve and lesser occipital nerve (LON), site V and VI, respectively, are also considered relevant trigger sites.^6^

The aim of this article was to describe the authors' technique to treat occipital migraine, while comparing our approach with the other currently available surgical options. Relevant anatomical issues and their implications in the surgical treatment of occipital migraine will also be reviewed.

## MATERIALS AND METHODS

### Relevant Anatomy

Sensitive innervation of occipital area is provided by the greater occipital nerve (GON), the LON, and the third occipital nerve (TON), the compression of which could potentially be the anatomical explanation of migraine symptoms.^7–11^

The GON arises from the medial branch of the C2 dorsal root. It runs toward the occipital region, running caudal to the inferior oblique muscle. Then, it crosses the semispinalis muscle, where it is possible to identify the deepest potential compression point of the nerve. According to previous anatomical investigations, GON is located 20.13 mm from the midline and 77.38 mm inferior from the occipital protuberance.^9^ The course of the nerve superiorly to nuchal line is variable.

Some studies reports that GON arches medially to the semispinalis muscle, piercing the fascial plane but this course is not constant as some other authors describe GON piercing the muscles of the occipital region: the semispinalis in the vast majority of cases (90% of cases), the inferior oblique, or the trapezius.^8,9^

Along the course of the GON, at least 6 points of compression can be found, which correspond to possible triggers for occipital migraine symptoms:^1^ the nerve passage through a tight fascial band between the obliquus capitis inferior and the semispinalis;^2^ its entrance into the deep fascia underlying the semispinalis or the muscle itself;^3,4^ its entrance into the semispinalis capitis and trapezius muscles;^5^ the piercing point of the nerve through the tendinous insertion of the trapezius into the nuchal line;^6^ the close neurovascular relationship between the GON and the occipital artery (OA).^8–11^ Indeed, anatomical studies demonstrated that a close relationship between the GON and the OA exists in more than 50% of individuals. This interaction could be an intertwining type of relationship when the artery coils around the nerve (70%) or it could be a single cross-type (30%) when the artery intercepts the nerve at a single point.^11,12^

Shimizu et al investigated also the anatomical relationship of the OA with the GON and identified a cross-point in proximity of the superior nuchal line where an indentation of the nerve was always present; however, histological examination failed to reveal any findings of nerve degeneration.^13^

Minor trigger sites in this area are the LON and to the third occipital nerve, which can be similarly compressed by fascial bands and branches of the OA, they seem to be the sources of pain for MH located in the lateral portion of the occipital and in the midportion occipital area, respectively.^14^

### Patient Selection

Patients eligible to undergo migraine deactivation surgery had to be diagnosed by a board-certified neurologist with migraine without aura with more than 15 days/mo of headache, lasting for more than 6 months, or chronic tension-type headache with more than 15 days/mo of headache, lasting for more than 6 months, or new daily persistent headache attacks with more than 15 days/mo of headache, lasting for more than 6 months. Cluster headache, episodic tension-type headache, and major psychiatric disease were regarded as major contraindications to perform the surgery. Furthermore, secondary migraine/headache as a consequence of other organic pathologies (ie, brain tumor, herniated disk of the neck) had to be ruled out through magnetic resonance or computer tomography investigations.

We firmly believe that clinical findings and physical examination are still the best way to identify trigger points' location. Patients with occipital MH typically experienced pain starting in the upper neck and occipital region at the point of exit of the GON from the semispinalis muscle.^15^ Manual compression of this site at the early stages of the migraine usually assuaged the pain, whereas at later stages, this point became tender. Patients could exhibit muscle tightness in the region. In addition, a history of whiplash or neck sprain might be present.^16^ If the patient's constellation of symptoms does not clearly indicate a specific trigger site, some authors suggest single site phased injection of botulinum toxin type A or a nerve block.^16^ However, a nerve block is only helpful when applied in patients who present with MHs at the time of the office visit, whereas botulinum toxin administration does not automatically exclude the suspected trigger point when the main cause of nerve compression is vascular in origin. Nerve blocks could be helpful; however, patients should be seen in the office during an MH attack.^15^ Therefore, we have never performed botulinum toxin type A injections neither nerve blocks as diagnostic tool, because it is the authors' experience that accurate history and careful physical examination can safely detect patient's trigger points. Indeed, even in patients reporting diffuse headache, the simple compression of the tender spot by the fingertip of the surgeon during physical examination could usually evoke pain confirming the diagnosis. We only used a handheld Doppler during physical examination because it proved to be an extremely useful tool in the identification of trigger points where the OA was irritating the nerve.^16^ Indeed, a positive Doppler signal at the location of pain identified by the patients strongly corroborated the diagnosis.

### Surgical Treatment

**Fig. 1.**
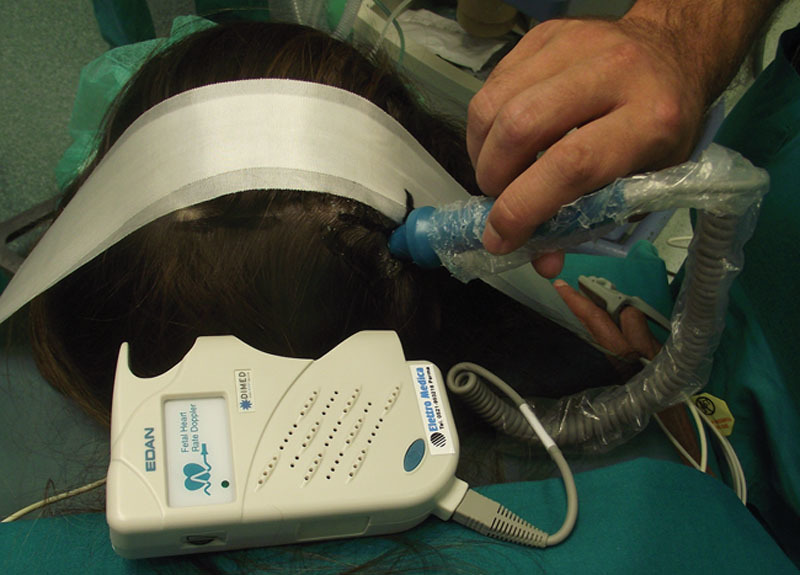
Arterial signal detected preoperatively by portable Doppler.

**Fig. 2.**
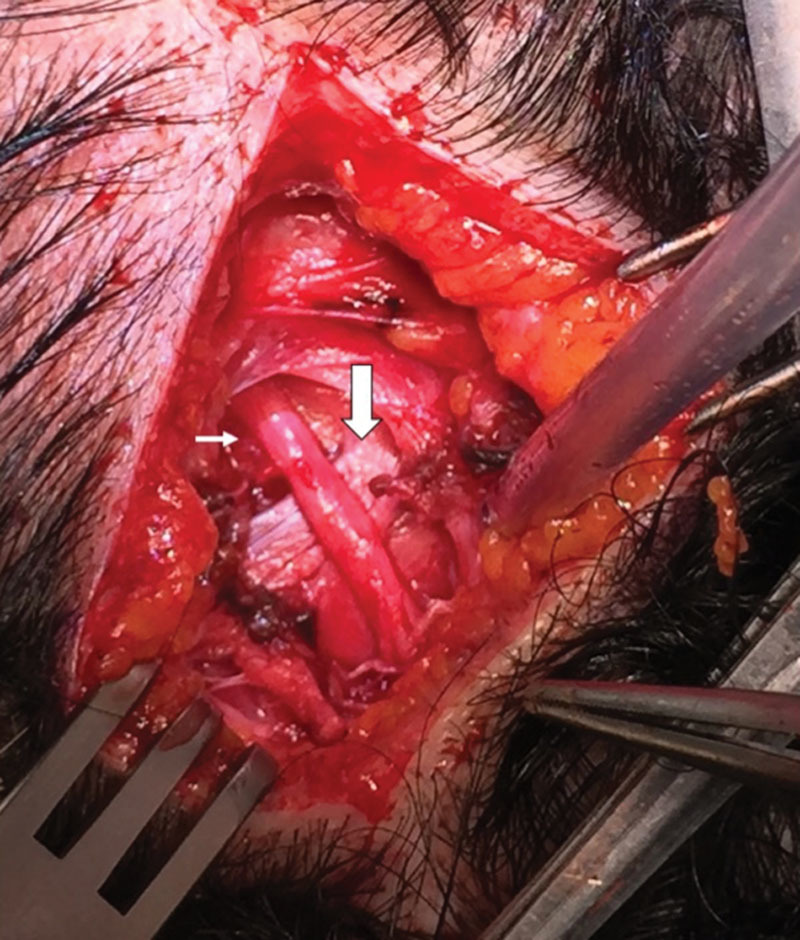
Close intersection of the great occipital nerve (big arrow) with the OA (small arrow).

**Fig. 3.**
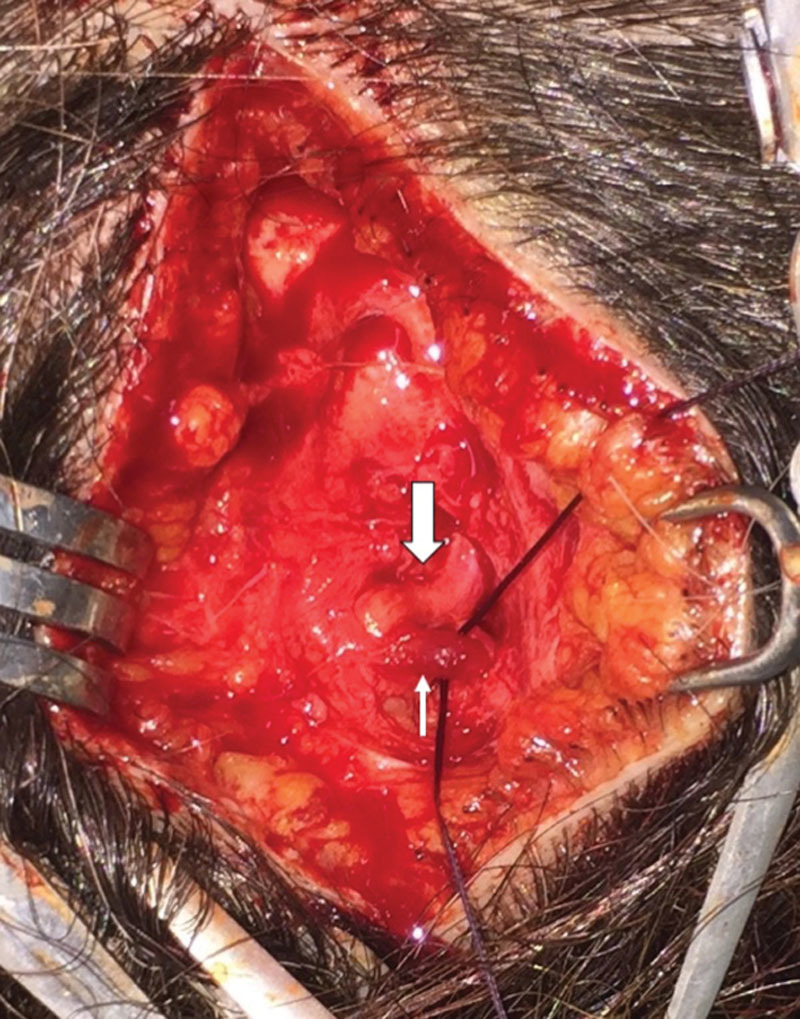
Ligation of OA (small arrow).

In accordance to our previously published works,^6,18–24^ we performed the occipital decompression with the patient prone, under local assisted anesthesia. After injecting 40 mL of diluted carbocaine 1% + 40-mL NaCl 0.9%, and 20-mL sodium bicarbonate 8.4%, 2 horizontal occipital scalp incisions of 5 cm in length were performed along the superior nuchal line bilaterally, at the location of arterial signal detected preoperatively by the handheld Doppler (Fig. 1). Underneath the subcutaneous tissue, an accurate dissection of occipital, trapezius, splenius capitis, and semispinalis capitis muscles allowed to identify the GON and vascular bundle (OA). When we found a dilated (or frankly aneurysmatic) OA in close connection with the GON (Fig. 2), we ligated the vessel without any other surgical maneuvers except for accurate hemostasis and skin closure (Fig. 3). In the remaining cases in which vascular compression was not found, we performed a conservative neurolysis of the GON and LON where the nerves were not isolated circumferentially to prevent complications and damages to the nerves as a consequence of reduced vascularization and following scar tissue formation. The occipital, trapezius, splenius capitis, and semispinalis capitis muscles were undermined along the nerves course until their emergence into the subcutaneous tissue. At the end of the procedure, after an accurate hemostasis, the cutaneous access was sutured with absorbable threads, without any drainage. No trichotomy was needed, and the scar from the incision will be hidden in the patient's hair. The total operative time was no longer than 90 minutes for bilateral incisions, but often it was less than 60 minutes when the relevant anatomical structures were easily identified.

Patients were permitted to resume ordinary activities in 1 week and heavy exercise in 3 weeks; they had to medicate every 2 days the surgical wound with betadine and could take a shower since the day after surgery. Patients were seen after initial recovery, at 1 month, and then every 3 months for 1 year.

Patients had to fill a daily headache diary and complete MH questionnaires assessing MH parameters following surgery. The same questionnaires were given preoperatively to assess changes in MH. Minimum follow-up was 12 months.

## RESULTS

We retrospectively reviewed the patients who underwent migraine deactivation surgery at our institution from June 2011 until February 2018; we performed MH decompression surgeries in 168 patients with either frontal, occipital, or temporal migraine trigger sites. Among them, 78 patients suffered from occipital migraine (58 bilateral and 20 unilateral).

In 56 patients with occipital migraine, we found a dilated OA in close connection with the GON and we ligated the vessel without any other surgical maneuvers. In 22 patients with occipital migraine, vascular compression was not found and we adopted a more conventional approach based on neurolysis of the GON and LON from muscles.

We gathered data from questionnaires completed before and after surgery. After a mean follow-up of 21 months (range: 12–67 months), patients with occipital migraine had positive response in 94.9% (86.8% complete relief and 8.1% significant improvement), and 5.1% did not get any better. As for the 56 patients who underwent dilated OA ligation, positive response 95.5% (90% complete relief and 5.5% significant improvement) and 4.5% did not get any better. As for the 22 patients who did not undergo OA ligation, we observed positive response in 91% (76% complete relief and 15% significant improvement) of the subjects whereas 9% did not get any better (Table 1). Statistical analysis by means of Chi-square test did not highlight a significant difference between the outcomes of the 2 groups.

**Table 1.** Gathered Data

	GON and/or LON Decompression	OAL	NTSM
Patients	78	56	22
Positive response	94.9%	95.5%	91%
Complete relief	86.8%	90%	76%
Partial relief	8.1%	5.5%	15%
No response	5.1%	3.5%	9%

NTSM, neurolysis at the trapezius and semispinalis muscles; OAL, OA ligation.

All the patients without improvement of the symptoms after OA ligation (4.5%) who suffered from unilateral occipital migraine had complete relief after contralateral secondary surgery.

Fourteen patients (8.3%) experienced secondary trigger point emergence following primary migraine surgery. Among these, 12 patients had 2 trigger points (10 occipital and frontal, 2 occipital and temporal) whereas 2 patients had all 3 trigger points.

### Adverse Events

Migraine surgery is regarded as a minimally invasive procedure; thus, no concerning side effects were reported. Minor and transient complications reported were intense itching (30%) and temporary anesthesia (mean duration 34 days) in all patients whereas postoperative infections, seromas, or hematomas were not observed. All patients who were refractory to surgery did not report worsening in their MH at any follow-up. Based on our data collected, secondary trigger point emergence following primary occipital migraine surgery occurred in 11.5% of patients. However, we routinely deactivate the main trigger site first, and then a second or third surgery was performed at the remaining sites 3 months after each surgery. MH recurrence may occur from 1 up to 3 months after surgery; thus, the result may be regarded as permanent only after the third postoperative month.^6,8,25,26^

## DISCUSSION

Almost 20 years have passed since Guyuron first introduced the concept of migraine surgery.^5^ He hypothesized that nerve compression at various trigger points was the origin of headache symptoms and further investigations were helpful to confirm his theory.^6,8,9,12^ Therefore, reported surgical treatment of occipital trigger site aimed at removing the potential compression points of the GON and the LON along their course throughout the semispinalis, the splenius, and the trapezius muscles to the subcutaneous tissue of the occipital scalp. According to Guyuron et al,^9^ Lin et al,^17^ Dash et al,^14^ and Lee et al,^27^ the currently adopted procedure for the treatment of the occipital trigger site, undertaken under general anesthesia, relied first on an incision in the occipital scalp followed by wide undermining, isolation of the GON from the subcutaneous tissue till its piercing point through the semispinalis muscle, extensive neurolysis of the GON, and ligation of the OA when the neurovascular relation was encountered in the subcutaneous tissue. Furthermore, a portion of both semispinalis and trapezius muscles, respectively, located medial and lateral to the GON, was removed. Subsequently, a caudally based subcutaneous flap was transposed between the GON and the muscle to avoid nerve impingement.

Guyuron et al^28^ reported a study of 195 patients who underwent occipital migraine surgery by semispinalis capitis resection and subcutaneous flap transposition to decompress the GON. Eighty-two percent of patients (n = 160) experienced greater than or equal to 50% occipital-specific MH index reduction, including 52% who experienced occipital-triggered MH elimination. One hundred sixty-eight patients (86.2%) observed at least 50% reduction in the number of migraine days. Migraine frequency (mean, 57.4% reduction), duration (mean, 36.9% reduction), and severity (mean, 58.6% reduction) were each significantly decreased postoperatively at the occipital site.

Lin et al^17^ reported their experience of 9 patients treated for occipital migraine by semispinalis capitis resection and subcutaneous flap transposition to decompress the GON. They observed a response rate greater than 90% and a complete resolution in 2 patients. One patient exhibited no response to the surgical intervention. Drug doses for headaches were reduced more than 50% in the remaining patients. The overall efficacy of occipital migraine surgery in their study was 88.8%.

Bovim et al^29^ treated 50 patients with occipital migraine by GON neurolysis at the trapezius muscle with a partial relief of 8% and no complete relief.

Magnússon et al^30^ in their study reported 13 occipital migraines treated by GON neurolysis at the trapezius muscle and at the semispinalis muscle with 72.2% of partial relief and no complete relief.

Larson et al^31^ in their study of 37 patients with occipital migraine who underwent semispinalis capitis resection and subcutaneous flap transposition to decompress the GON observed partial relief in 34.3% of patients and complete relief in 45.5% of patients.

Chmielewski et al^7^ treated 55 patient by OA ligation with 41.8% of partial relief and 38.2% of complete relief, and 115 patients by semispinalis capitis resection and subcutaneous flap transposition with 27% of partial relief and 64.3% of complete relief.

Ducic et al^32^ reported 190 patients with occipital migraine treated by GON neurolysis at the trapezius muscle and at the semispinalis muscle furthermore the semispinalis capitis muscle resection with 37.1% of partial relief and 43.4% of complete relief.

Because the exact location and the structures involved in nerve compressions are still a matter of investigation, most authors performed personal modifications of the Guyuron's technique based on their findings and personal belief. The main differences between our procedure and the ones described above were that, in most cases, extensive undermining or muscular resection was not necessary and no flap was transposed with the purpose of covering isolated nerves.^18^

Based on our experience, the ligation of OA when dilated compared with the GON e/or LON neurolysis at the trapezius and semispinalis muscle allowed a greater percentage of complete relief (90% versus 76%; *P* > 0.5) with a minor percentage of partial relief (5.5% versus 15%; *P* > 0.5). The percentage nonresponse patients after OA ligation resulted inferior than the “neurolysis” group (4.5% versus 9%; *P* > 0.5). Furthermore, because of 4.5% included only unilateral migraine, after the secondary contralateral surgery the percentage of nonresponse patients following OA ligation was zero. This finding could be due to anastomosis between contralateral vessels, making monolateral ligation ineffective. However, other factors could be involved and further investigations will be necessary to address the optimal surgical procedure for monolateral migraine.

Our strategy allowed us to achieve the highest rate of surgical success when compared with other ones presented. Furthermore, we did not observe some of the minor complication commonly reported in literature as incisional cellulitis, transient mild incisional alopecia or hair thinning (probably due to the subcutaneous flap harvest with the involuntary damage to the hair bulbs), and lasting neck stiffness that has a reported incidence ranging from 1% to 5% and might be a consequence of the extensive undermining and muscles removal.^6,8,18–25^

Our more conservative approach was the consequence of our experience and belief that the OA played a pivotal role in triggering occipital migraine by compressing the GON in most cases. The success rate of our occipital migraine surgery technique suggests that the close relationship between artery and nerve was, in most cases, essential. Therefore, surgical treatment should attempt to remove any possible site of nerve compression along the OA. In author's experience, this aim was achieved by OA ligation without any other surgical maneuvers when was dilated (or frankly aneurysmatic) in close connection with the GON. Indeed, Shimizu et al^13^ reported a constant and close relationship between OA and GON nerve. An indentation was always present, and the cross-point, although no histological findings, was evident. These findings support the hypothesis that the cross-point between OA and occipital nerve is an important landmark for the surgeon who attempts to relieve the compression on the nerve.

## CONCLUSIONS

Occipital migraine is a common and debilitating condition that can be treated successfully with surgery. Its anatomical basis is far from being completely understood but it seems evident that nerve compression in the occipital area is the main causative agent. According to our experience, a dilated OA was usually responsible for nerve compression, mostly the GON. Clinical outcome of our surgical procedure based on artery ligation seemed to prove the validity of this hypothesis. In addition, we also described a minimally invasive approach, thus reducing operative time and complication rate.

## ACKNOWLEDGMENTS

This study did not need any ethical approval because it did not describe experimental studies on either humans or animals. All participants provided informed consent.
